# Apoptosis signal-regulating kinase 1 mediates denbinobin-induced apoptosis in human lung adenocarcinoma cells

**DOI:** 10.1186/1423-0127-16-43

**Published:** 2009-05-01

**Authors:** Chen-Tzu Kuo, Bing-Chang Chen, Chung-Chi Yu, Chih-Ming Weng, Ming-Jen Hsu, Chien-Chih Chen, Mei-Chieh Chen, Che-Ming Teng, Shiow-Lin Pan, Mauo-Ying Bien, Chung-Hung Shih, Chien-Huang Lin

**Affiliations:** 1 Graduate Institute of Medical Sciences, College of Medicine, Taipei Medical University, Taipei, Taiwan, ROC; 2 School of Respiratory Therapy, College of Medicine, Taipei Medical University, Taipei, Taiwan, ROC; 3 Department of Pharmacology, College of Medicine, Taipei Medical University, Taipei, Taiwan, ROC; 4 National Institute of Chinese Medicine, Taipei, Taiwan, ROC; 5 Department of Microbiology and Immunology, College of Medicine, Taipei Medical University, Taipei, Taiwan, ROC; 6 Pharmacological Institute, College of Medicine, National Taiwan University, Taipei, Taiwan, ROC; 7 Taipei Medical University-Municipal Wang-Fang Hospital, Taipei, Taiwan, ROC; 8 Taipei Medical University-Shuang-Ho Hospital, Taipei County, Taiwan, ROC

## Abstract

In the present study, we explore the role of apoptosis signal-regulating kinase 1 (ASK1) in denbinobin-induced apoptosis in human lung adenocarcinoma (A549) cells. Denbinobin-induced cell apoptosis was attenuated by an ASK1 dominant-negative mutant (ASK1DN), two antioxidants (N-acetyl-L-cysteine (NAC) and glutathione (GSH)), a c-Jun N-terminal kinase (JNK) inhibitor (SP600125), and an activator protein-1 (AP-1) inhibitor (curcumin). Treatment of A549 cells with denbinobin caused increases in ASK1 activity and reactive oxygen species (ROS) production, and these effects were inhibited by NAC and GSH. Stimulation of A549 cells with denbinobin caused JNK activation; this effect was markedly inhibited by NAC, GSH, and ASK1DN. Denbinobin induced c-Jun phosphorylation, the formation of an AP-1-specific DNA-protein complex, and Bim expression. Bim knockdown using a *bim *short interfering RNA strategy also reduced denbinobin-induced A549 cell apoptosis. The denbinobin-mediated increases in c-Jun phosphorylation and Bim expression were inhibited by NAC, GSH, SP600125, ASK1DN, JNK1DN, and JNK2DN. These results suggest that denbinobin might activate ASK1 through ROS production to cause JNK/AP-1 activation, which in turn induces Bim expression, and ultimately results in A549 cell apoptosis.

## Background

Denbinobin (5-hydroxy-3,7-dimethoxy-1,4-phenanthraquinone) is purified from several *Dendrobium *or *Ephemerantha *(Orchidaceae) species, such as *D. nobile *[1], *D. moniliforme *[2], and *E. lonchophylla *[3,4]. It has been shown that denbinobin possesses many biological effects such as antioxidation, antiplatelet aggregation, and anti-inflammation. It was also demonstrated to induce cell death in colon adenocarcinoma (COLO 205) cells, ovary adenocarcinoma (SK-OV-3) cells, human leukemia (K562) cells, promyelocytic leukemia (HL60) cells, and human lung adenocarcinoma (A549) cells [1,5-7]. Previous studies demonstrated that alterations in tubulin polymerization and Bcr-Abl activity are involved in denbinobin-induced human K562 leukemia cell death [5]. Recently, we demonstrated that Akt inactivation, followed by Bad activation, mitochondrial dysfunction, the release of mitochondrial molecules such as cytochrome c, second mitochondria-derived activator of caspase (Smac), and apoptosis-inducing factor (AIF), and caspase 3 activation contributes to denbinobin-induced A549 cell apoptosis [6]. However, the precise molecular mechanism of denbinobin-induced A549 cell apoptosis has not been fully delineated.

Apoptosis signal-regulating kinase 1 (ASK1) is a multifunctional serine/threonine protein kinase that participates in diverse physiological processes, including cell differentiation and apoptosis [8,9]. ASK1 has been reported to be activated by many stress signals, including H_2_O_2_, tumor necrosis factor (TNF)-α, and endoplasmic reticular stress [10-12]. However, the role of ASK1 in denbinobin-induced A549 cell apoptosis is still unknown. Bim is a member of the "BH3-only proteins", a subgroup of Bcl-2 apoptotic regulators which contain only one of the bcl-2 homologous regions (BH3). In response to apoptotic stimuli, BH3-only proteins are translocated to mitochondrial membranes from other cellular compartments where they interfere with the function of antiapoptotic Bcl-2 family members, leading to apoptotic cell death [13,14].

In the present study, we explored the roles of ASK1 in denbinobin-induced cell death in human lung adenocarcinoma (A549) cells. Our data demonstrate for the first time that denbinobin might activate ASK1 through reactive oxygen species (ROS) production to cause JNK/AP-1 activation, which in turn induces Bim expression, and ultimately results in A549 cell apoptosis.

## Materials and methods

### Materials

Denbinobin was kindly provided by Dr. Chien-Chih Chen (National Research Institute of Chinese Medicine, Taipei, Taiwan). The methods of extraction and isolation of denbinobin were previously reported [3], and the purity exceeded 98% based on a high-performance liquid chromatographic (HPLC) analysis. Dulbecco's modified Eagle's medium (DMEM)/Ham's F-12, fetal calf serum (FCS), penicillin/streptomycin, OptiMEM, Lipofectamine plus™ reagent, and TRIzol reagent were purchased from Invitrogen (Carlsbad, CA). Antibodies specific for phospho-JNK1/2 (Thr^183^/Tyr^185^), JNK1/2, and phospho-Akt (Ser^473^) were purchased from New England Biolabs (Beverly, MA). The α-tubulin antibody was purchased from Novus Biologicals (Littleton, CO). Protein A/G beads, antibodies specific for JNK1, phospho-c-Jun, c-Jun, c-Fos, Akt1/2, and GAPDH, as well as horseradish peroxidase-conjugated anti-mouse and anti-rabbit antibodies were purchased from Santa Cruz Biotechnology (Santa Cruz, CA). Anti-mouse immunoglobulin G (IgG)-conjugated alkaline phosphatase was purchased from Jackson Immuno Research Laboratories (West Grove, PA). The enhanced chemiluminescence detection agent was purchased from PerkinElmer Life Sciences (Boston, MA). 4-Nitro blue tetrazolium (NBT) and 5-bromo-4-chloro-3-indolyl-phosphate (BCIP) were purchased from Boehringer Mannheim (Mannheim, Germany). All materials for sodium dodecylsulfate polyacrylamide gel electrophoresis (SDS-PAGE) were obtained from Bio-Rad (Hercules, CA). SP600125 was purchased from Calbiochem-Novabiochem (San Diego, CA). 2',7'-Dichlorodihydrofluorescein diacetate (H2DCFDA) was purchased from Molecular Probes (Eugene, OR). ASK1DN, JNK1DN, and JNK2DN were kindly provided by Dr. Mei-Chieh Chen (Taipei Medical University, Taipei, Taiwan). All materials for agarose gel electrophoresis and [γ-^32^P] ATP were obtained from GE Healthcare (Little Chalfont, UK). The RNAi-Ready pSIREN-RetroQ-ZsGreen vector was purchased from BD Biosciences-Clontech (Palo Alto, CA). All materials for sqRT-PCR were obtained from Protech Technology (Taipei, Taiwan). N-Acetyl-L-cysteine (NAC), glutathione (GSH), propidium iodide (PI), dithiothreitol (DTT), phenylmethylsulphonyl fluoride (PMSF), pepstatin A, leupeptin, SDS, 3-(4,5-dimethyl-thiazol-2-yl)-2,5-diphenyltetrazolium (MTT), and other chemicals were obtained from Sigma (St. Louis, MO).

### Cell culture

A549 cells, a human pulmonary type II epithelial adenocarcinoma cell line, were obtained from the American Type Culture Collection and cultured in DMEM/Ham's F-12 nutrient mixture with 10% FCS and antibiotics (100 U/ml penicillin and 100 μg/ml streptomycin). Cells were cultured at 37°C in a humidified 5% CO_2 _atmosphere.

### Flow cytometric analysis

A549 cells were cultured in 10-cm Petri dishes. After reaching confluence, cells were transfected with different plasmid DNAs for 6 h or pretreated with specific inhibitors for 30 min as indicated followed by treatment with 20 μM denbinobin for additional 24 h. After treatment, cells were harvested and washed twice with phosphate-buffered saline (PBS, 137 mM NaCl, 2.7 mM KCl, 4.3 mM Na_2_HPO_4_, and 1.5 mM KH_2_PO_4_; pH 7.4), and re-suspended in ice-cold 70% ethanol at -20°C overnight. Cells were washed for 5 min with 0.4 ml phosphate-citric acid buffer (pH 7.8) containing 50 mM Na_2_HPO_4_, 25 mM citric acid, and 0.1% Triton X-100 and subsequently stained with 1.5 ml PI staining buffer containing 0.5% Triton X-100, 10 mM PIPES, 100 mM NaCl, 2 mM MgCl_2_, 0.1 U/ml RNase A, and 25 μg/ml PI for 30 min in the dark before the flow cytometric analysis. Samples were analyzed by FACScan and the Cellquest program (Becton Dickinson, San Jose, CA).

### Cell viability assay

Cell viability was measured by a previously described colorimetric MTT assay [15]. Briefly, cells (2 × 10^5 ^cells/well) were cultured in 24-well plates and transfected with different plasmid DNAs for 6 h or pretreated with specific inhibitors for 30 min as indicated followed by incubation with 20 μM denbinobin for another 24 h. After various treatments, 1 mg/ml MTT was added to the culture plates and incubated at 37°C for an additional 4 h. Then cells were lysed in 500 μl dimethyl sulfoxide. The absorbance at 550 nm was measured on a microplate reader. Each experiment was performed in triplicate and repeated at least three times.

### Plasmid DNA transfection

A549 cells were seeded at a density of 2 × 10^5 ^cells/ml into 12-well plates. Cells were transfected on the following day with the Lipofectamine plus™ reagent containing 1 μg/well of pcDNA (mock), ASK1DN, JNK1DN, JNK2DN, pSiREN-NC, or pSiREN-*bim *cDNA in Opti-MEM for 6 h. At the end of transfection, the medium was aspirated and replaced with fresh culture medium for 24 h. Cells were treated with 20 μM denbinobin for various time periods dependent on different experiments before harvesting. To determine the transfection efficiency, cells were transfected with 1 μg of pEGFP, a green fluorescence protein (GFP) expression vector for 24 h. After treatment, the medium was aspirated and replaced with fresh DMEM/Ham's F12 containing 10% FBS for another 24 h. Cells were observed under inverted laser scanning confocal microscopy (Olympus). The transfection efficacy was defined as the percentage of cells expressing GFP. The transfection rate of GFP was about 33% (data not shown).

### Measurements of ASK1 and JNK kinase activity

A549 cells were seeded in 6-cm dishes. After reaching confluence, cells were treated with 20 μM denbinobin for 10, 30, 60, or 120 min. After treatment, cells were lysed with lysis buffer containing 20 mM Tris-HCl (pH 7.5), 1 mM MgCl_2_, 125 mM NaCl, 1% Triton X-100, 1 mM PMSF, 10 μg/ml leupeptin, 10 μg/ml aprotinin, 25 mM β-glycerophosphate, 50 mM NaF, and 100 μM Na_3_VO_4_, and centrifuged at 4°C and 12,000 × *g *for 30 min. The supernatant was then immunoprecipitated with a specific antibody against ASK1 or JNK1/2 in the presence of A/G-agarose beads overnight. The beads were washed three times with lysis buffer and two times with kinase buffer containing 20 mM HEPES (pH 7.4), 20 mM MgCl_2_, and 2 mM DTT. The kinase reactions were performed by incubating immunoprecipitated beads with 20 μl of kinase buffer supplemented with substrate (50 μg/ml myelin basic protein (MBP) for ASK1 activity or 50 μg/ml c-Jun-GST fusion protein for JNK1/2 activity), 20 μM ATP, and 3 μCi of [γ-^32^P] ATP at 30°C for 30 min. The reaction mixtures were analyzed by 15% SDS-PAGE followed by autoradiography.

### ROS generation assay

Intracellular ROS formation was measured using the fluorogenic probe, H2DCFDA (Molecular Probes, Eugene, OR). Briefly, A549 cells were loaded with 10 μM H_2_DCFDA for 30 min at 37°C in the dark. After incubation, cells were treated with 20 μM denbinobin for the indicated time intervals, or pretreated with specific inhibitors as indicated followed by denbinobin. Cells then harvested, and ROS generation was measured by FACScan flow cytometry to detect the log of the mean fluorescence intensity (MFI) with an acquisition of fluorescence channel 1 (FL1). A minimum number of 10,000 events was collected and analyzed by the Cellquest program.

### Immunoblot analysis

An immunoblot analysis was performed as previously described [16]. A549 cells were cultured in 6-cm dishes. After reaching confluence, cells were treated with 20 μM denbinobin for the indicated time intervals, or pretreated with specific inhibitors, or transiently transfected with pcDNA, ASK1DN, JNK1DN, or JNK2DN for 6 h followed by denbinobin. After incubation, cells were washed twice with ice-cold PBS and solubilized in extraction buffer containing 10 mM Tris (pH 7.0), 140 mM NaCl, 3 mM MgCl_2_, 2 mM PMSF, 5 mM DTT, 0.5% NP-40, 0.01 mg/ml aprotinin, 0.01 mg/ml leupeptin, 1 mM benzamidine, and 1 mM Na_3_VO_4_. Protein concentrations of cell lysates were determined by the Bradford protein assay (Bio-Rad). Equal amounts of protein (60 μg) in each sample were boiled in SDS sample loading buffer, and then fractionated on SDS-PAGE before being blotted onto a polyvinylidene difluoride (PVDF) membrane. Blots were then incubated in 150 mM NaCl, 20 mM Tris, and 0.02% Tween (pH 7.4) containing 5% non-fat milk. Proteins were visualized by specific primary antibodies and then incubated with alkaline phosphatase- or horseradish peroxidase-conjugated second antibodies. After washing with PBS, blots were developed using NBT/BCIP or an enhanced chemiluminescence kit according to the vendor's instructions before exposure to photographic film.

### Preparation of nuclear extracts and the electrophoretic mobility shift assay (EMSA)

Cells were grown in 6-cm dishes. After reaching confluence, cells were treated with 20 μM denbinobin for the indicated time intervals. The nuclear protein fractions were then prepared as described previously [17]. Briefly, cells were washed with ice-cold PBS, and then centrifuged. The cell pellet was resuspended in hypotonic buffer (10 mM HEPES, 10 mM KCl, 0.5 mM DTT, 10 mM aprotinin, 10 mM leupeptin, and 20 mM PMSF) for 15 min on ice, and vortexed for 10 sec. The nuclei were collected by centrifugation at 15,000 × g for 1 min. The pellet containing nuclei was resuspended in hypertonic buffer (20 mM HEPES (pH 7.6), 25% glycerol, 1.5 mM MgCl_2_, 4 mM EDTA, 0.05 mM DTT, 20 mM PMSF, 10 mM aprotinin, and 10 mM leupeptin) for 30 min on ice. The supernatants containing the nuclear proteins were collected by centrifugation at 15,000 × g for 30 min and then stored at -80°C.

A double-stranded oligonucleotide probe containing the recombinant AP-1 consensus sequence (5'-TTC CGG CTG ACT CAT CAA GCG-3'; Promega) was end-labeled with [γ-^32^P] ATP using the T4 polynucleotide kinase. The nuclear extract (2.5~5 μg) was incubated with 1 ng of a ^32^P-labeled AP-1 probe (50,000~75,000 cpm) in 10 μl of binding buffer containing 1 μg poly(dI-dc), 15 mM HEPES (pH 7.6), 80 mM NaCl, 1 mM EDTA, 1 mM DTT, and 10% glycerol at 30°C for 25 min. DNA/nuclear protein complexes were separated from the DNA probe by electrophoresis on 6% polyacrylamide gels. The gels were vacuum-dried and subjected to autoradiography with an intensifying screen at -80°C. For competition experiments, 1 ng of the labeled oligonucleotide was mixed with 50 ng of AP-1 or NF-κB unlabeled competitor oligonucleotides prior to protein addition. For the supershift experiments, 4 μg of the anti-c-Jun or anti-c-Fos antibody was mixed with the nuclear extract proteins.

### Interference of bim expression

To generate siRNAs targeting *bim *mRNA for degradation, we produced the pSiREN-*bim *plasmid to suppress bim expression. To create pSiREN-*bim*, artificial complementary oligonucleotides were annealed and cloned into the pSIREN-RetroQ-ZsGreen vector (BD Biosciences Clontech, San Diego., CA): the sense oligonucleotide sequence for *bim *was 5'-GATCCGTTCTGAGTGTGACCGAGAttcaagagaTCTCGGTCACACTCAGAACTTTTTTG-3'; and the antisense oligonucleotide sequence for *bim *was 3'-GCAAGACTCACACTGGCTCTaagttctctAGAGCCAGTGTGAGTCTTGAAAAAACTTAA-5'. A negative control RNAi (pSiREN-NC) was also constructed by cloning custom-synthesized oligonucleotides into the pSIREN-RetroQ-ZsGreen vector. The accuracies of the pSiREN-*bim *and pSiREN-NC plasmids were confirmed based on the sequencing analysis.

### RNA isolation and semiquantitative (sq)RT-PCR

A549 cells were seeded in 6-cm dishes. After reaching confluence, cells were treated with 20 μM denbinobin or 5 mM H_2_O_2 _for the indicated time intervals, or pretreated with specific inhibitors (1 mM NAC, 100 μM GSH, or 10 μM SP600125) for 30 min, or transiently transfected with pcDNA, ASK1DN, JNK1DN, or JNK2DN for 6 h followed by denbinobin. Total RNA was purified using the TRIzol reagent (Invitrogen) following the manufacturer's protocol and quantified by spectrophotometry at a 260 nm wavelength. RNA was reverse-transcribed, and the cDNA product was amplified by PCR using 500 ng of specific forward and reverse primers. β-Actin was normalized in parallel in the same samples. PCR conditions included pre-incubation at 95°C for 5 min followed by 40 amplification cycles (denaturation at 95°C for 1 min, annealing at 53°C for 1 min, elongation at 72°C for 1 min, and a final extension for 10 min at 72°C). Forward and reverse primers for bim were designed for all isoforms except for bim-γ: sense, 5'-AAG CAA CCT TCT GAT GTA-3'; antisense, 5'-CAA TGC ATT CTC CAC ACC AG-3'. The sense primer of β-actin was 5'-GAC TAC CTC ATG AAG ATC CT-3'; and the reverse primer of β-actin was 5'-CCA CAT CTG CTG GAA GGT GG-3'. PCR products were visualized by UV-illuminated agarose gel electrophoresis.

### Statistical analysis

Results are presented as the mean ± SEM from at least three independent experiments. One-way analysis of variance (ANOVA), followed by Bonferroni's multiple-range tests when appropriate, was used to determine the statistical significance of the difference between the means. A *p *value of < 0.05 was considered statistically significant.

## Results

### ASK1 mediates denbinobin-induced A549 cell apoptosis

**Figure 1:**
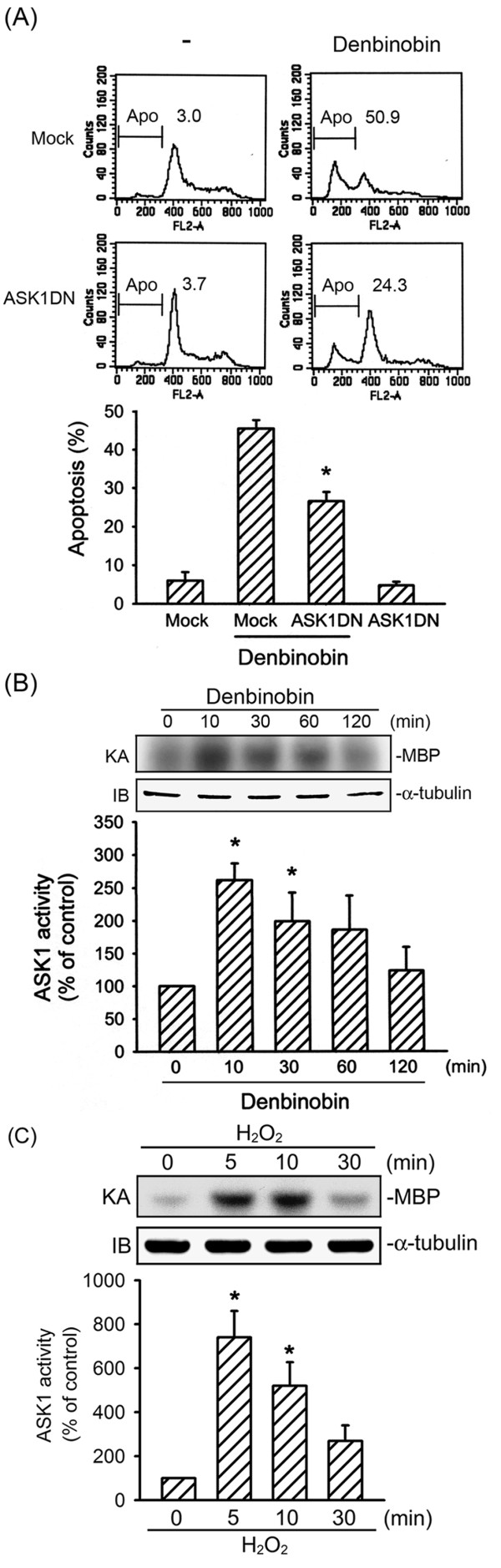
**ASK1 activation is involved in denbinobin-induced apoptosis in A549 cells**. (A) A549 cells were transiently transfected with pcDNA (mock) or ASK1DN for 6 h. Following transfection, cells were replaced with fresh culture medium for 24 h and treated with vehicle or 20 μM denbinobin for another 24 h. The percentage of apoptotic cells was then determined using flow cytometric analysis of PI-stained cells as described in "Materials and methods". Each column represents the mean ± S.E.M. of at least three independent experiments. *p < 0.05, compared to the mock transfection group in the presence of denbinobin. Cells were treated with 20 μM denbinobin (B) or 5 mM H_2_O_2 _(C) for the indicated time intervals. Cell lysates were then immunoprecipitated with the anti-ASK1 antibody, and the kinase activity was measured as described in "Materials and methods". Equal loading in each lane is reflected by similar intensities of α-tubulin. Compiled results are shown in the lower panel. Each column represents the mean ± S.E.M. of at least three independent experiments. * *p *< 0.05, compared to the control group without denbinobin or H_2_O_2 _treatment.

ASK1 activation is a pivotal mechanism in a broad range of cell death paradigms [12]. To explore whether ASK1 activation contributes to denbinobin-induced A549 cell apoptosis, cells were transiently transfected with the ASK1DN prior to denbinobin treatment. As shown in Fig. 1A, the percentage of PI-stained cells in the apoptotic region (Apo, sub-G0/G1 peak, subdiploid peak) was significantly increased following 20 μM denbinobin treatment, compared to the vehicle-treated group. Denbinobin-induced A549 cell apoptosis was attenuated by 48.1 ± 5.2% by transfection with ASK1DN (Fig. 1A). To further elucidate whether ASK1 activation is involved in the signaling cascade of denbinobin-induced A549 cell apoptosis, ASK1 kinase activity was measured after denbinobin exposure. Treatment of A549 cells with 20 μM denbinobin increased ASK1 kinase activity. This response peaked at 10 min and had returned to the basal level within 2 h (Fig. 1B). Similarly, 5 mM H_2_O_2_, a potent ASK1 stimulator [18], caused an increase in ASK1 kinase activity in a time-dependent manner (Fig. 1C). These results suggest that ASK1 activation participates in denbinobin-induced apoptosis in A549 cells.

### Involvement of ROS generation in denbinobin-induced A549 cell apoptosis

**Figure 2:**
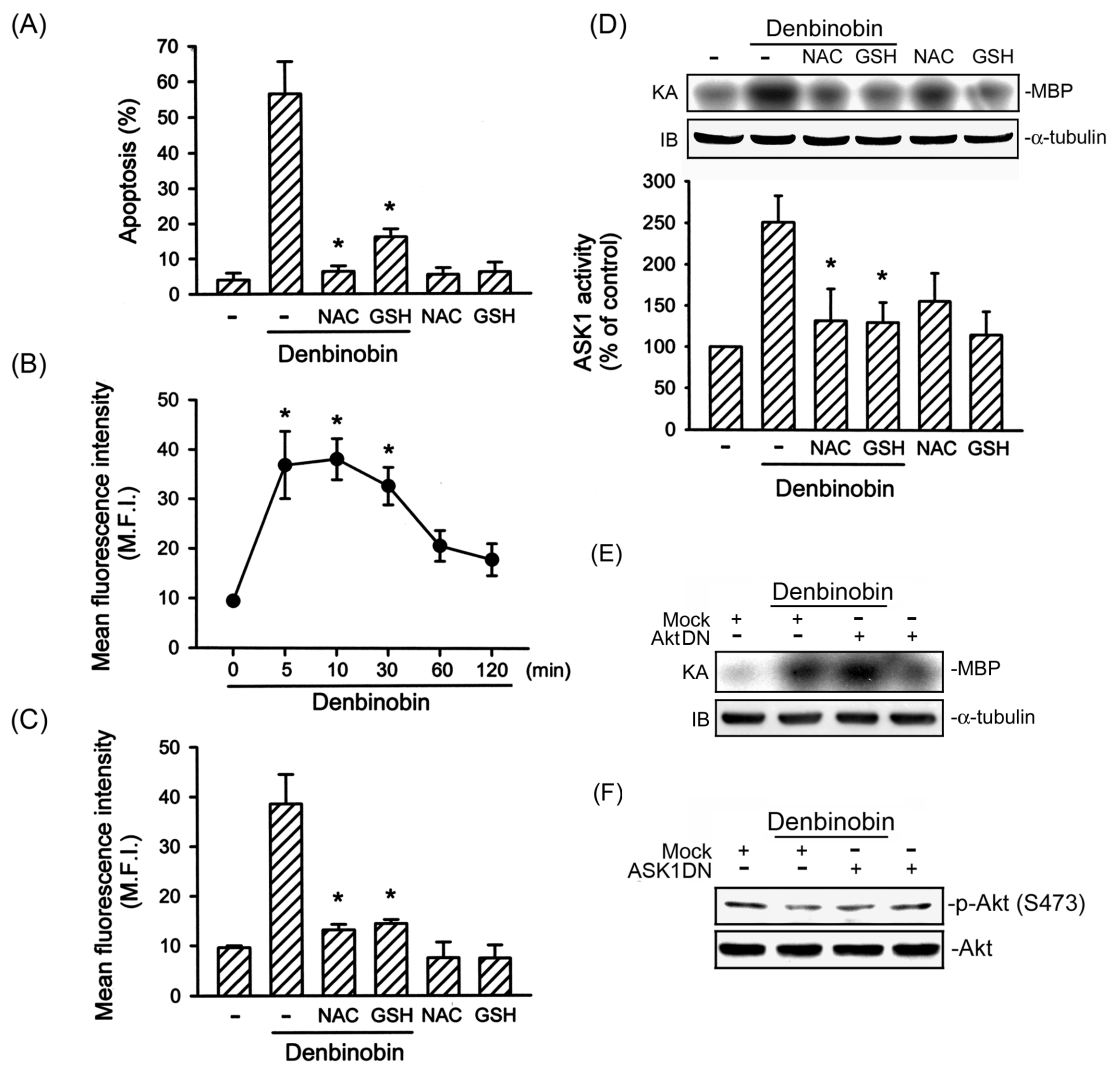
**ROS generation mediates denbinobin-induced apoptosis in A549 cells**. (A) Following pretreatment with NAC (1 mM) or GSH (100 μM) for 30 min, cells were incubated with the vehicle or 20 μM denbinobin for another 24 h. Cells then were harvested, and apoptosis was analyzed by flow cytometry as described in "Materials and methods". Each column represents the mean ± S.E.M. of at least three independent experiments performed in triplicate. * *p *< 0.05, compared to the group treated with denbinobin. (B) Cells were treated with 20 μM denbinobin, and DCF fluorescence was monitored by flow cytometry for up to 120 min as described in "Materials and methods". Results are plotted as the mean fluorescence intensity (MFI) ± S.E.M. of five independent experiments. * *p *< 0.05, compared to the control group. (C) Cells were pretreated with NAC (1 mM) or GSH (100 μM) for 30 min prior to denbinobin (20 μM) stimulation for 10 min. ROS generation was detected by H2DCFDA using flow cytometry. Data are represented as the MFI ± S.E.M. of three independent experiments. * *p *< 0.05, compared to the group treated with denbinobin. (D) Cells were pretreated with NAC (1 mM) or GSH (100 μM) for 30 min before treatment with 20 μM denbinobin for another 10 min. ASK1 kinase activity was then measured. Equal loading in each lane is reflected by similar intensities of α-tubulin. Compiled results are shown in the lower panel. Each column represents the mean ± S.E.M. of at least three independent experiments. * *p *< 0.05, compared to the control group in the presence of denbinobin. Cells were transiently transfected with pcDNA (mock), AktDN (E) or ASK1DN (F) for 6 h. Following transfection, cells were replaced with fresh culture medium for 24 h and treated with vehicle or 20 μM denbinobin for another 10 min (for ASK1 kinase activity) or 6 h (for Akt phosphorylation). Cell lysates were then prepared and subjected to ASK1 kinase activity or Akt phosphorylation as described in "Materials and methods". Equal loading in each lane is demonstrated by similar intensities of α-tubulin or Akt1/2, respectively. Compiled results are shown in the lower panel. Typical traces are representative of two experiments with similar results.

Previous report indicated that ROS play an important role in apoptotic signaling pathway [19]. To explore the role of ROS in denbinobin-induced A549 cell apoptosis, the antioxidants, NAC and GSH, were used. As shown in Fig. 2A, pretreatment of A549 cells with 1 mM NAC and 100 μM GSH markedly attenuated denbinobin-induced A549 cell apoptosis by 95.4 ± 2.8% and 77.0 ± 4.1%, respectively. Moreover, treatment of cells with 20 μM denbinobin caused a time-dependent increase in ROS production with a maximum effect at 5~10 min after denbinobin treatment. However, after 60 min of treatment with denbinobin, the response had decreased (Fig. 2B). Furthermore, denbinobin-induced ROS formation was almost completely inhibited by treatment with 1 mM NAC or 100 μM GSH (Fig. 2C). These results suggest that denbinobin caused intracellular ROS generation, and the oxidative stress further contributed to A549 cell apoptosis. Previous studies showed that ROS might activate ASK1 through oxidizing thioredoxin (Trx) and glutaredoxin [20-22]. We next speculated whether ROS generation results in ASK1 activation in denbinobin-mediated apoptosis. As illustrated in Fig. 2D, the denbinobin-induced increase in ASK1 activity was markedly inhibited by 1 mM NAC and 100 μM GSH by 79.5 ± 15.1% and 80.1 ± 9.6%, respectively. Our previous study has shown that denbinobin-induced A549 cell apoptosis involving Akt inactivation [6]. Next, we investigated whether Akt is involved in denbinobin-induced ASK1 activity. When A549 cells transfected with 1 μg AktDN did not affect denbinobin-induced ASK1 activity (Fig. 2E). Furthermore, we examined whether ASK1 is involved in denbinobin-induced Akt inactivation. As shown in Fig. 2F, transfection of A549 cells with 1 μg ASK1DN did not affect denbinobin-induced Akt Ser473 dephosphorylation. These results suggest that denbinobin-induced Akt inactivation and ASK1 activation are through two independent signal pathways.

### JNK is involved in denbinobin-induced A549 cell apoptosis

**Figure 3:**
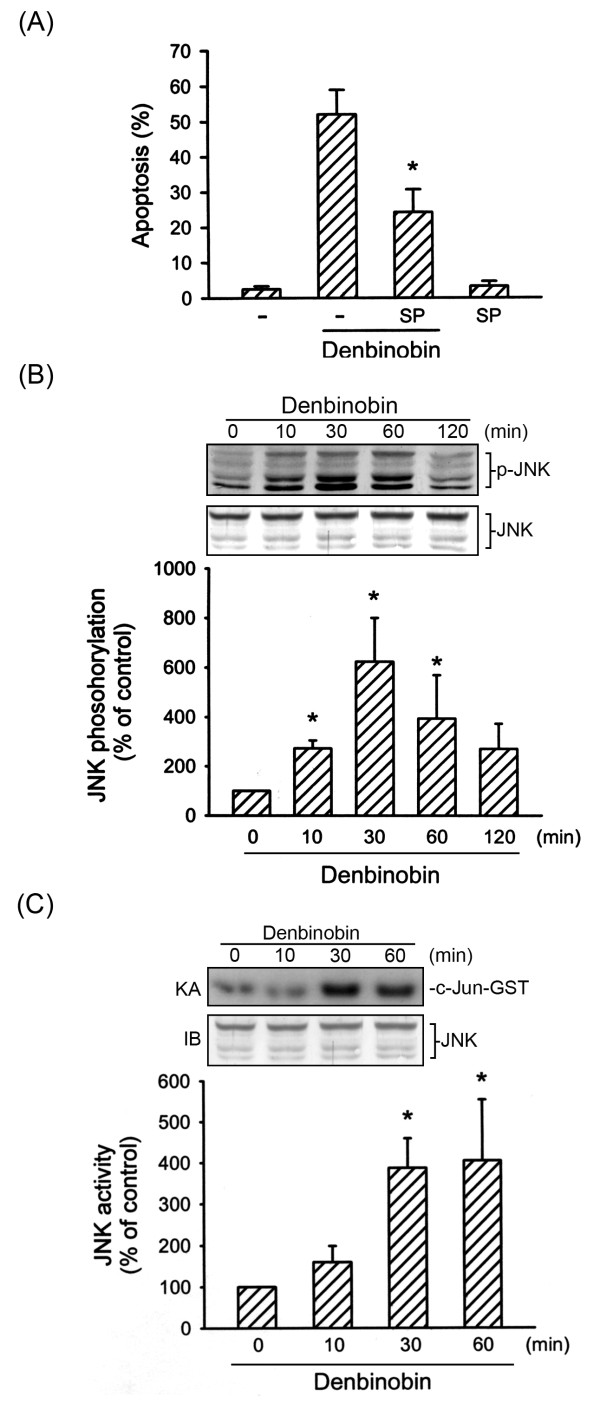
**Involvement of JNK activation in denbinobin-induced apoptosis in A549 cells**. (A) Cells were pretreated with SP600125 (10 μM) for 30 min before incubation with 20 μM denbinobin for 24 h, and then apoptosis was detected using flow cytometry of PI-stained cells as described in "Materials and methods". Each column represents the mean ± SEM of at least three independent experiments. *p < 0.05, compared to the denbinobin group. Cells were treated with 20 μM denbinobin for the indicated time intervals. Cell lysates were then prepared and subjected to JNK phosphorylation (B) or JNK kinase activity (C) as described in "Materials and methods". Equal loading in each lane is demonstrated by similar intensities of JNK. Compiled results are shown in the lower panel. Each column represents the mean ± S.E.M. of at least three independent experiments. * *p *< 0.05, compared to the control group.

**Figure 4:**
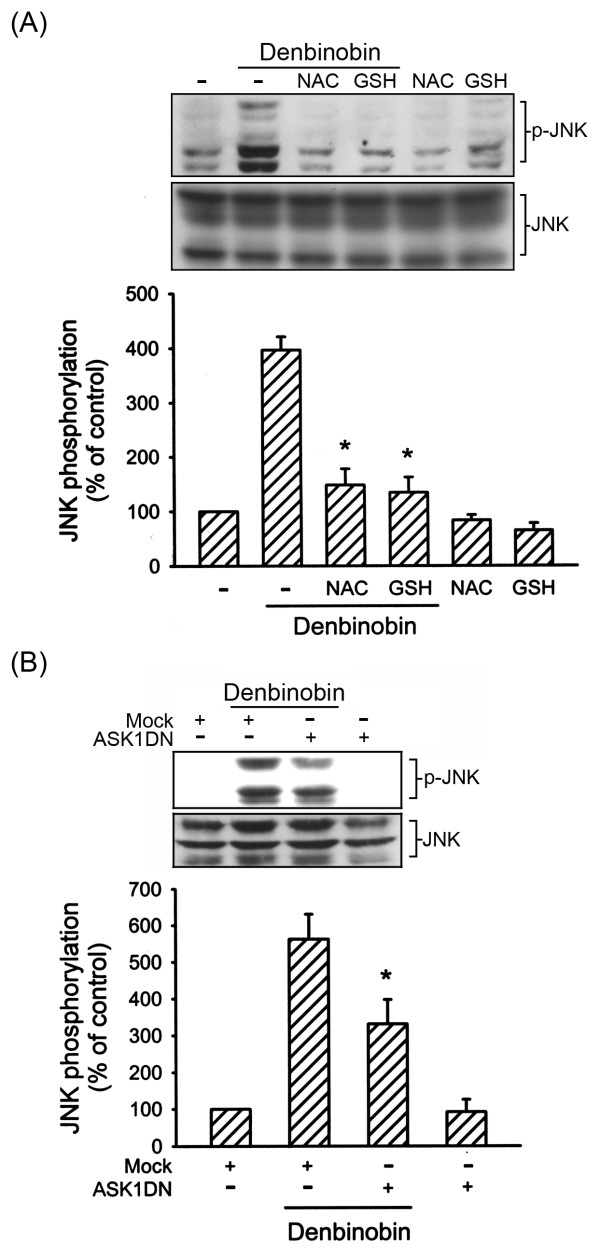
**Involvement of ROS and ASK1 in denbinobin-induced JNK activation in A549 cells**. Cells were pretreated with 1 mM NAC or 100 μM GSH (A) or transiently transfected with pcDNA or ASK1DN for 6 h (B) before incubation with 20 μM denbinobin for 30 min. Cells were then harvested and subjected to immunoblotting for JNK phosphorylation as described in "Materials and methods". Equal loading in each lane is shown by the similar intensities of JNK. Compiled results are shown in the lower panel. Each column represents the mean ± S.E.M. of at least three independent experiments. * *p *< 0.05, compared to the denbinobin group.

ASK1 belongs to the MAPKKK family and activates the JNK and p38MAPK pathways via MKK4/7 and MKK3/6, respectively [11]. In the present study, we focused on the role of the JNK signaling cascade in denbinobin-induced A549 cell apoptosis. We examined whether JNK signaling events are involved in denbinobin-induced A549 cell apoptosis. The specific JNK inhibitor, SP600125 (10 μM), markedly attenuated denbinobin-induced A549 cell apoptosis by 56.0 ± 12.3% (Fig. 3A). We next examined whether denbinobin was able to activate JNK1/2. Results from Fig. 3B illustrate that 20 μM denbinobin time-dependently increased JNK1/2 phosphorylation, with a maximum effect at 30 min of denbinobin exposure. The protein level of JNK1/2 was not affected by the presence of denbinobin (Fig. 3B, bottom panel). In parallel, using the c-Jun-GST fusion protein as the JNK1/2 substrate, a time-dependent increase in JNK1/2 activity was also observed in denbinobin-treated A549 cells (Fig. 3C). To determine the relationships among ROS, ASK1, and JNK in the denbinobin-mediated signaling pathway, we determined the effects of two antioxidants (NAC and GSH) and ASK1DN on denbinobin-induced JNK activation. As shown in Fig. 4A, pretreatment of A549 cells for 30 min with 1 mM NAC and 100 μM GSH markedly inhibited denbinobin-induced JNK1/2 activation. Neither of these treatments had any effect on the protein level of JNK1/2 (Fig. 4A, bottom panel). Furthermore, transfection of A549 cells with ASK1DN significantly reduced denbinobin-induced JNK1/2 activation by 50.1 ± 11.7% (Fig. 4B). However, ASK1DN did not affect the protein level of JNK1/2 (Fig. 4B, bottom panel). Taken together, these findings suggest that the ROS-ASK1 cascade is required for denbinobin-induced JNK1/2 activation in A549 cells.

### AP-1 is involved in denbinobin-induced A549 cell apoptosis

**Figure 5:**
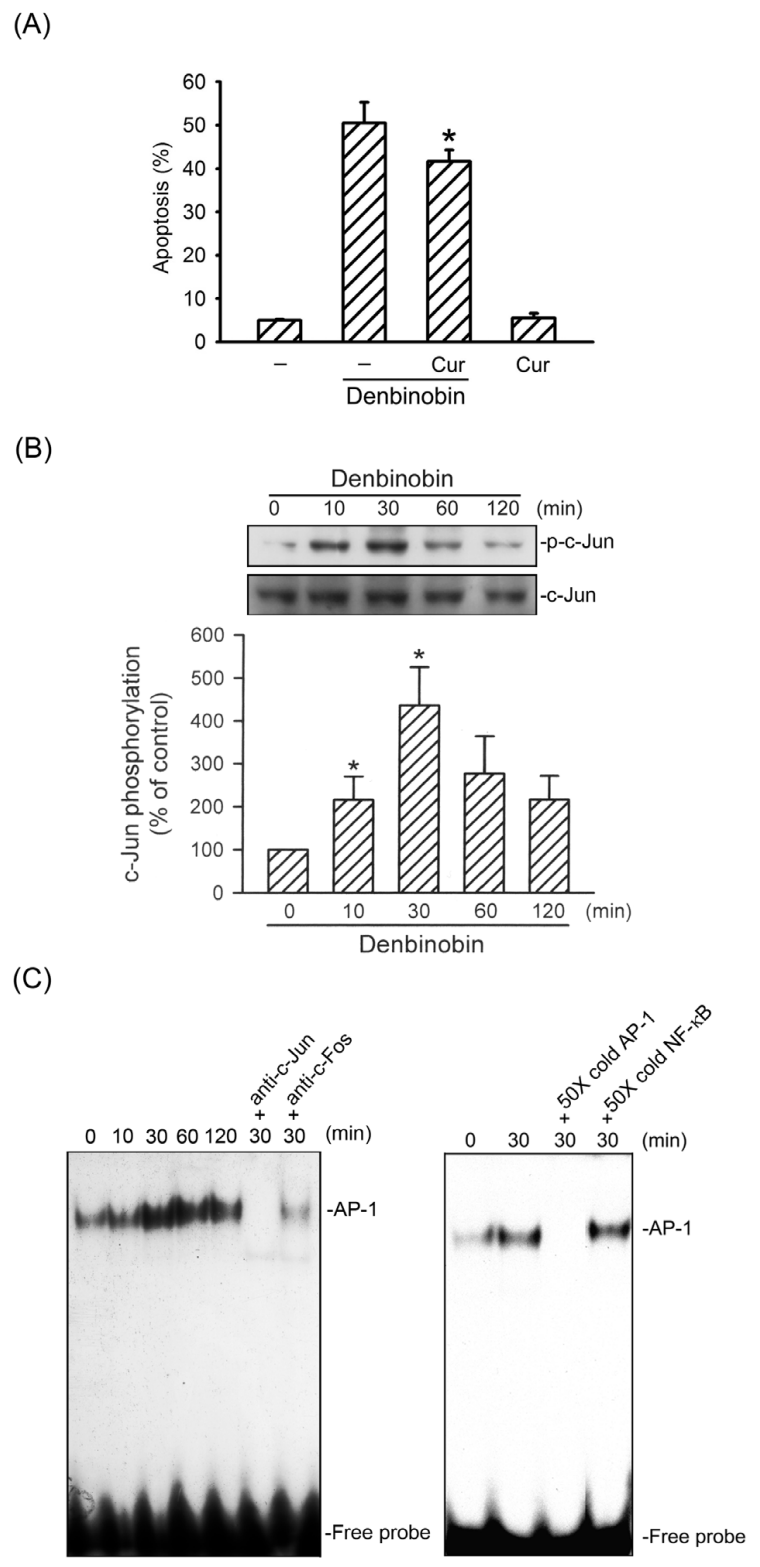
**Involvement of AP-1 activation in denbinobin-induced apoptosis in A549 cells**. (A) Cells were pretreated with curcumin (1 μM) for 30 min before incubation with 20 μM denbinobin for another 24 h, and then apoptosis was detected using flow cytometry of PI-stained cells as described in "Materials and methods". Each column represents the mean ± SEM of at least three independent experiments. *p < 0.05, compared to the denbinobin group. (B) Cells were treated with 20 μM denbinobin for 10, 30, 60, and 120 min. Cells were then harvested and subjected to immunoblotting with an anti-phospho-c-Jun antibody as described in "Materials and methods". Equal loading in each lane is shown by the similar intensities of c-Jun. Compiled results are shown in the lower panel. Each column represents the mean ± S.E.M. of at least three independent experiments. * *p *< 0.05, compared to the control group. (C) Cells were incubated with 20 μM denbinobin for various time intervals. Following incubation, the nuclear protein fraction was prepared, and AP-1-specific DNA protein complex formation was analyzed by an EMSA as described in "Materials and methods". The antibodies of c-Jun and c-Fos were incubated prior to detecting the specificity of AP-1 binding activity. Fifty-fold concentrations of unlabeled AP-1 or NF-κB oligonucleotides (50× cold) were used for the competition experiment. Data shown are representative of three independent experiments with similar results.

**Figure 6:**
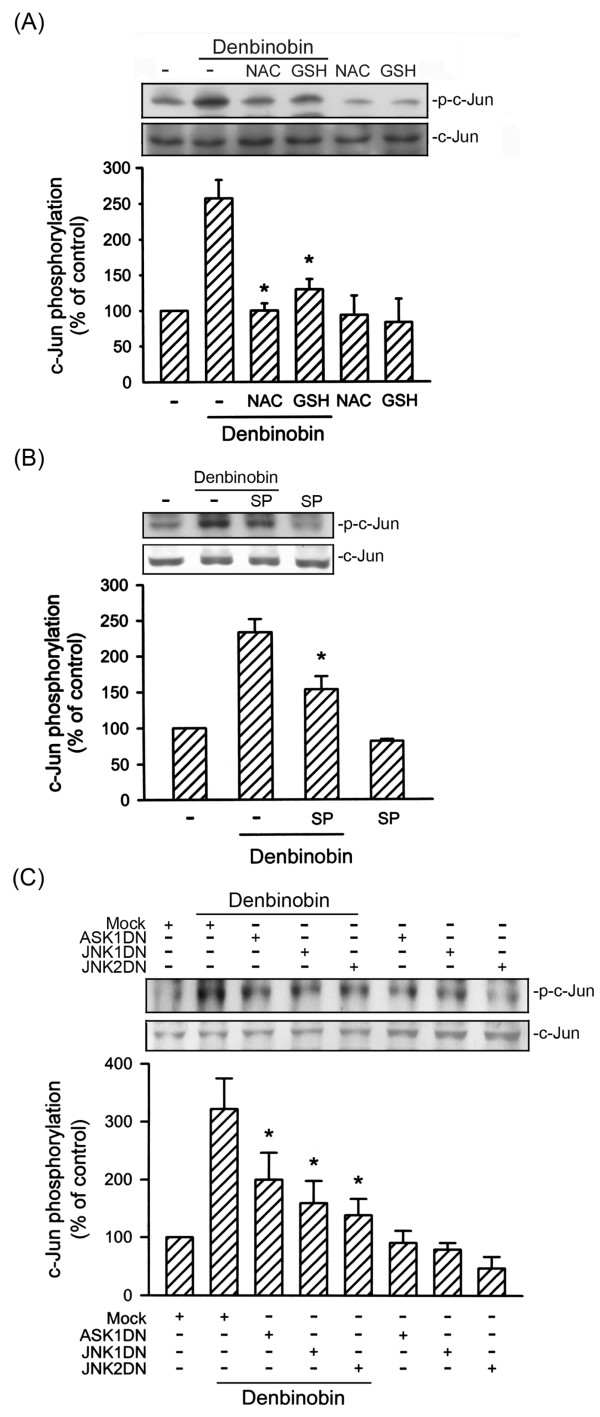
**Involvement of ROS, ASK1, and JNK in denbinobin-induced c-Jun phosphorylation in A549 cells**. Cells were pretreated with 1 mM NAC, 100 μM GSH (A), or 10 μM SP600125 (B), or transiently transfected with pcDNA, ASK1DN, JNK1DN, or JNK2DN for 6 h (C). After treatment, cells were treated with vehicle or 20 μM denbinobin for 30 min. Cells were then harvested for immunoblotting to assess the level of c-Jun phosphorylation as described in "Materials and methods". Equal loading in each lane is reflected by approximately similar intensities of the c-Jun bands. Compiled results are shown in the lower panel. Each column represents the mean ± S.E.M. of at least three independent experiments. * *p *< 0.05, compared to the denbinobin group. * *p *< 0.05, compared to the control group (A and B) or the pcDNA (mock) group (C) in the presence of denbinobin.

We next wished to determine whether AP-1 is involved in denbinobin-induced cell apoptosis by using the AP-1 inhibitor curcumin. As shown in Fig. 5A, denbinobin-induced cell apoptosis was attenuated by pretreatment of cells with 1 μM curcumin by 21 ± 5% (n = 3). Moreover, 1 μM curcumin inhibited the decrease of cell viability after exposure to 20 μM denbinobin by 42 ± 10% by MTT test (data not shown). The transcription factor, AP-1, is composed of heterodimeric protein complexes comprising a protein family group such as c-Jun and c-Fos. Activation of c-Jun requires a phosphorylation process manipulated by activated JNK [23]. Therefore, we measured c-Jun phosphorylation after denbinobin treatment. Treatment of A549 cells with 20 μM denbinobin caused a time-dependent increase in c-Jun phosphorylation. This response reached a maximum at 30 min and had declined after 120 min treatment with denbinobin (Fig. 5B). The protein level of c-Jun was not affected by the presence of denbinobin (Fig. 5B, bottom panel). The nuclear extracts of A549 cells with or without denbinobin treatment were subjected to EMSA using AP-1-specific oligonucleotides as the probes. As shown in Fig. 5C, AP-1-specific DNA-protein complex formation time-dependently increased with a maximum effect at 30 min of denbinobin treatment. However, after 120 min of treatment with denbinobin, the intensities of these DNA-protein complexes had decreased (Fig. 5C). Formation of the DNA-protein complex was completely reduced by the addition of 50× cold AP-1 consensus DNA oligonucleotides. However, 50× cold NF-κB consensus DNA oligonucleotides did not affect AP-1-specific DNA-protein complex formation (Fig. 5C). These results indicated that the DNA-protein interactions were AP-1 sequence specific. To identify the specific subunits involved in the formation of the AP-1 complex after denbinobin stimulation, EMSA was also performed in the presence of specific antibodies against c-Jun or c-Fos. As shown in Fig. 5C, pretreatment of nuclear extracts with a specific antibody against either c-Jun or c-Fos reduced the AP-1-specific DNA-protein binding activity, demonstrating that the components of the AP-1 heterodimer are c-Jun and c-Fos. Furthermore, we determined whether ROS, ASK1, and JNK mediated denbinobin-induced c-Jun activation. As shown in Fig. 6, denbinobin-induced c-Jun phosphorylation was markedly attenuated in cells pretreated for 30 min with 1 mM NAC, 100 μM GSH, and 10 μM SP600125 by 99.9 ± 3.9%, 81.6 ± 5.6%, and 59.9 ± 7.8%, respectively (Fig. 6A, B). Moreover, transfection of A549 cells with ASK1DN, JNK1DN, and JNK2DN all inhibited denbinobin-induced c-Jun phosphorylation (Fig. 6C). Based on these results, we suggest that the ROS-ASK1-JNK1/2 cascade occurs upstream of c-Jun phosphorylation in the denbinobin-induced signaling pathway.

### Bim upregulation is involved in denbinobin-induced apoptosis

**Figure 7:**
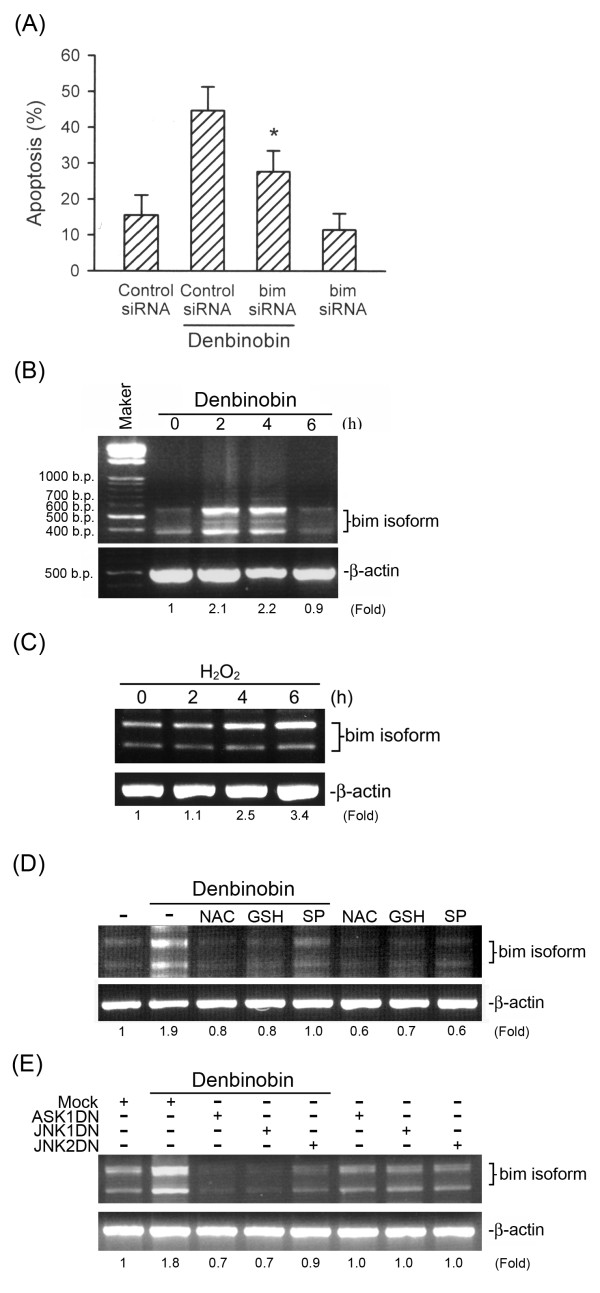
**Bim expression is involved in denbinobin-induced apoptosis in A549 cells**. (A) Following transfection with control siRNA or *bim *siRNA for 6 h, cells were treated with vehicle or 20 μM denbinobin for another 24 h. The DNA content was then analyzed by flow cytometry of PI-stained cells as described in "Materials and methods". Each column represents the mean ± S.E.M. of at least three independent experiments. * *p *< 0.05, compared to the control siRNA group in the presence of denbinobin. Cells were incubated with 20 μM denbinobin (B) or 5 mM H_2_O_2 _(C) for the indicated intervals, pretreated with 1 mM NAC, 100 μM GSH, or 10 μM SP600125 (D), or transiently transfected with pcDNA, ASK1DN, JNK1DN, or JNK2DN for 6 h (E). Cells were then treated with vehicle or 20 μM denbinobin for 2 h. Following treatment, RNA was collected to assess bim expression by semiquantitative RT-PCR as described in "Materials and methods". Samples were normalized for β-actin intensities. Typical traces are representative of three experiments with similar results.

To investigate whether silencing Bim interrupts denbinobin-induced A549 cell apoptosis, cells were transiently transfected with Bim-specific siRNA (pSiREN-*bim*) following DNA content analysis by flow cytometry. As shown in Fig 7A, cells receiving pSiREN-*bim *displayed a reduced proportion of denbinobin-induced cell apoptosis by 44.5 ± 12.9%, suggesting that Bim upregulation mediates A549 cell apoptosis induced by denbinobin. Next, we examined whether denbinobin treatment increased expression of Bim mRNA determined by sqRT-PCR. As shown in Fig. 7B, exposure of A549 cells to 20 μM denbinobin resulted in Bim mRNA expression. A maximal response was reached after 2~4 h of treatment, and had decreased to the basal level after 6 h of treatment. Similarly, 5 mM H_2_O_2 _induced Bim mRNA expression in a time-dependent manner (Fig. 7C). Furthermore, pretreatment of A549 cells with 1 mM NAC, 100 μM GSH, and 10 μM SP600125 all inhibited denbinobin-mediated Bim mRNA expression (Fig. 7D). Moreover, denbinobin-induced Bim mRNA expression was also markedly inhibited by transfection with ASK1DN, JNK1DN, and JNK2DN (Fig. 7E). These results suggest that the ROS-ASK1-JNK cascade is required for denbinobin-induced Bim expression in A549 cells.

## Discussion

Our previous study showed that Akt inactivation, followed by Bad activation, mitochondrial dysfunction, caspase 3 activation, and AIF release, contributes to denbinobin-induced apoptosis in human lung adenocarcinoma (A549) cells. However, the precise molecular mechanism responsible for the apoptotic action of denbinobin remains to be fully characterized. The findings of this study demonstrated that activation of the ROS-ASK1-JNK signaling cascade followed by AP-1 activation and Bim expression contributes to denbinobin-induced apoptosis in A549 cells.

Activated ASK1 plays a critical role in regulating diverse physiological processes, including cell differentiation and apoptosis, by stimulating downstream signaling events including activation of MKK4/7 and JNK in sequence [24,25]. JNK is thought to play a role in AP-1-mediated transcriptional activation of the target genes [26,27]. However, whether the ASK1-JNK-AP-1 signaling pathway participates in denbinobin-induced A549 cell apoptosis has not been demonstrated. In this study, we found that treatment of A549 cells with denbinobin caused sequential activations of ASK1, JNK, c-Jun, and AP-1, and that the dominant-negative mutants of ASK1, JNK1, and JNK2, and the inhibitors of JNK and AP-1 attenuated denbinobin-induced cell apoptosis in A549 cells. The dominant-negative mutant of ASK1 suppressed denbinobin-induced JNK1/2 activation. Moreover, denbinobin-induced c-Jun phosphorylation was inhibited by the dominant-negative mutants of ASK1, JNK1, and JNK2, and the JNK inhibitor. These results suggest that denbinobin might activate ASK1, causing JNK and c-Jun activation, which in turn induces AP-1 activation, and ultimately leads to A549 cell apoptosis.

ASK1 activity is regulated by interactions with various proteins. When free of oxidative stress, the inactive form of ASK1 is phosphorylated at the serine at residue 967 and is suppressed by interactions with its repressors, such as thioredoxin (Trx), glutaredoxin (Grx), and the 14-3-3 protein. Previous studies showed that ROS might activate ASK1 through oxidizing thioredoxin (Trx) and glutaredoxin [20-22]. Recent reports also indicated that ASK1 is response to oxidative stress such as H_2_O_2 _through decreasing phosphorylation at serine 967 parallel to increasing the dissociation with inhibitory proteins leading to activation of the apoptotic function [20,28]. In this study, we found that denbinobin induced ROS formation, and that two antioxidants (NAC and GSH) inhibited denbinobin-induced ASK1 activation, JNK activation, c-Jun phosphorylation, and cell apoptosis. In addition, treatment of A549 cells with H_2_O_2 _induced ASK1 activation. These findings suggest that ROS may play a pivotal role in denbinobin-induced ASK1 activation and subsequent signaling events.

Bcl-2 family proteins regulate mitochondria-dependent apoptosis with the balance of the anti- and proapoptotic members arbitrating life-or-death decisions. Bim, a proapoptotic member of the Bcl-2 family, causes apoptosis by disrupting mitochondrial integrity. *bim gene expression is induced by *AP-1 in response to selected stress signals [29,30]. In the present study, we found that denbinobin induced Bim expression and that Bim siRNA attenuated denbinobin-induced A549 cell apoptosis, suggesting that Bim expression is causally related to denbinobin-induced A549 cell apoptosis. Furthermore, denbinobin-induced Bim expression was inhibited by antioxidants, a JNK inhibitor, and the dominant-negative mutants of ASK1, JNK1, and JNK2. Similarly, treatment of cells with H_2_O_2 _induced ASK1 activation. Thus, it is plausible that denbinobin activates the ROS-ASK1-JNK cascade causing Bim expression and subsequent cell apoptosis. Recent reports have indicated that in addition to Bim, BH3-only members of the Bcl-2 family, such as Bad, also participate in denbinobin-induced A549 cell apoptosis [6]. These observations explain, at least in part, why blocking the ASK1 signaling cascade did not completely abolish denbinobin-induced A549 cell apoptosis.

## Conclusion

**Figure 8:**
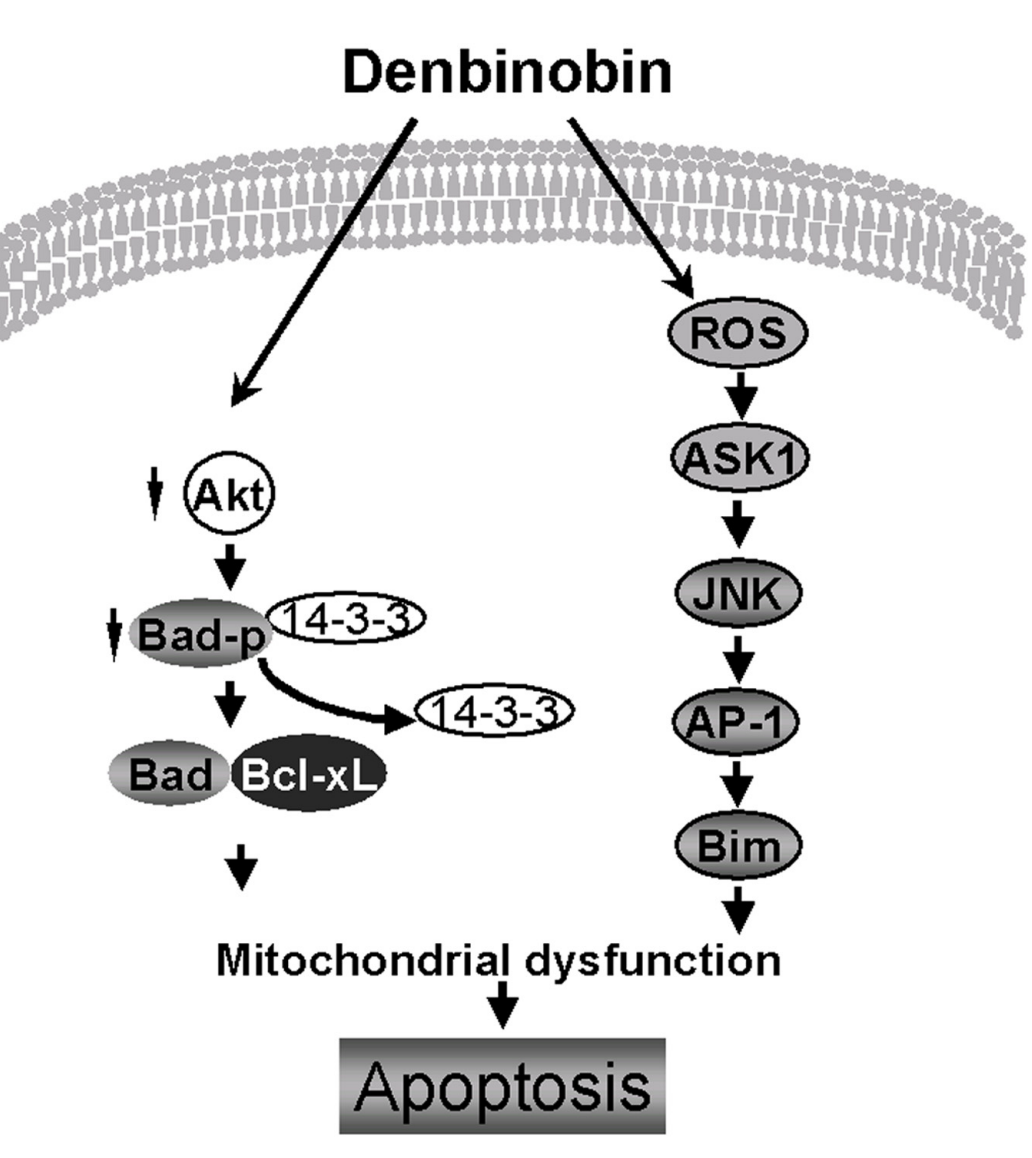
**Schematic summary of the apoptotic pathway involved in denbinobin-induced A549 cell apoptosis**. Denbinobin caused Akt inactivation, leading to Bad dephosphorylation, mitochondrial dysfunction, and subsequent cell apoptosis. Denbinobin also activated ASK1 through ROS generation to cause JNK/AP-1 activation, which in turn induced Bim expression, ultimately resulting in A549 cell apoptosis.

In conclusion, results from the present study demonstrate for the first time that denbinobin-induced A549 cell apoptosis is involved at least in part with activation of the ROS-ASK1-JNK-c-Jun signaling cascade to induce AP-1 activation and Bim expression. The present study, together with our previous report delineates, in part, the signaling pathways involved in denbinobin-induced A549 cell apoptosis. Figure 8 is a schematic representation of the signaling pathway of denbinobin-induced cell apoptosis in human lung adenocarcinoma cells. Thus, denbinobin may be useful as a potential template for the development of better chemopreventive and/or chemotherapeutic agents.

## Abbreviations

AP-1: activator protein-1; ASK1: apoptosis signal-regulating kinase 1; BCIP: 5-bromo-4-chloro-3-indolyl-phosphate; Bim: Bcl-2-interacting mediator; BSA: bovine serum albumin; DMEM: Dulbecco's modified Eagle's medium; DTT: dithiothreitol; EMSA: electrophoretic mobility shift assay; FCS: fetal calf serum; GSH: glutathione; H2DCFHDA: 2',7'-dichlorodihydrofluorescein diacetate; HEPES: 4-(2-hydroxy-ethyl)-1-piperazineethanesulfonic acid; JNK: c-Jun N-terminal kinase; MTT: 3-(4,5-dimethylthiazol-2-yl)-2,5-diphenyl tetrazolium bromide; NAC: N-acetyl-L-cysteine; NBT: 4-nitro blue tetrazolium; NP-40: Nonident P-40; PBS: phosphate-buffered saline; PI: propidium iodide; PMSF: phenylmethylsulphonyl fluoride; ROS: reactive oxygen species; SDS: sodium dodecylsulfate; siRNA: short interfering RNA; SOD: superoxide dismutase.

## Competing interests

The authors declare that they have no competing interests.

## Authors' contributions

CTK participated in the design of the study, performed major experiments and the data interpretation. BCC designed the experiments and interpreted the data. CCY and CMW participated in part of the experiments. MJH participated in the design of the study and interpreted the data. CCC participated in the design of the study and provided the experimental materials. MCC constructed the plasmids. CMT and SLP participated in the design of the study and data interpretation. MYB and CHS participated in data interpretation and manuscript improvement. CHL conceived of the study, and participated in its design and coordination. All authors read and approved the final manuscript.
